# CTLs: Killers of intracellular bacteria

**DOI:** 10.3389/fcimb.2022.967679

**Published:** 2022-10-26

**Authors:** Li Tian, Wei Zhou, Xianwei Wu, Zhuannan Hu, Lei Qiu, Huiyong Zhang, Xue Chen, Shaoyan Zhang, Zhenhui Lu

**Affiliations:** ^1^ Institute of Respiratory Diseases, Longhua Hospital, Shanghai University of Traditional Chinese Medicine, Shanghai, China; ^2^ School of Life Sciences, Shanghai University, Shanghai, China

**Keywords:** cytotoxic T lymphocytes, CD4 CTLs, CD8 CTLs, intracellular bacteria, perforin, SMAP, Fas-FasL, cytokines

## Abstract

Many microbial pathogens have evolved a range of capabilities to evade host immune defense mechanisms and to survive and multiply in host cells. The presence of host intracellular bacteria makes it difficult for specific antibodies to function. After the intracellular bacteria escape the attack of the innate immune system, such as phagocytes, they survive in cells, and then adaptive immunity comes into play. Cytotoxic T lymphocytes (CTLs) play an important role in eliminating intracellular bacteria. The regulation of key transcription factors could promote CD4^+^/CD8^+^ T cells to acquire cytolytic ability. The TCR-CD3 complex transduces activation signals generated by TCR recognition of antigen and promotes CTLs to generate multiple pathways to kill intracellular bacteria. In this review, the mechanism of CD4/CD8 CTLs differentiation and how CD4/CD8 CTLs kill intracellular bacteria are introduced. In addition, their application and prospects in the treatment of bacterial infections are discussed.

## 1 Introduction

The innate immune system is activated first after pathogenic microorganisms invade the organism. Phagocytic cells in the body rapidly reach the site of infection, recognizing pathogen-associated molecular patterns (PAMPs) through surface pattern recognition receptors (PRRs). Pathogenic microorganisms are engulfed by phagocytes into phagosomes, which subsequently fuse with lysosomes to form phagolysosomes (Mitchell et al., 2016). The phagocytosed pathogenic microorganisms are killed by phagocytosis within an environment of low pH and lack of nutrients and antimicrobial activity (e.g., oxidative, nitrosative, and proteolytic stress). By contrast, many pathogenic microorganisms develop the ability to evade digestion and elimination by macrophages, thus surviving for a long time within the host cell (Baxt et al., 2013), hence called intracellular bacteria.

Intracellular bacteria are divided into two categories: facultative intracellular bacteria and obligate intracellular bacteria (Casadevall and Fang, 2020). Facultative intracellular bacteria, including *Mycobacterium tuberculosis (MTB)*, *Listeria monocytogenes (Listeria)*, *Bacillus leprosy*, *Salmonella typhi*, *Brucella*, and *Legionella pneumophila*, could not only reproduce and survive in host cells but also grow in inanimate medium *in vitro*. They also mostly cause chronic infection. Meanwhile, obligate intracellular bacteria, including *Rickettsia*, *Kirkus*, and *Chlamydia*, could only grow and reproduce in the living cells of the host (Otten et al., 2018). Intracellular pathogens have a range of virulence factors and a complex network of regulatory factors, such as membrane dynamics, actin cytoskeleton, phosphatidylinositol metabolism, intracellular transport, and immune defense mechanisms (Voss and Rahman, 2021), that control host cellular processes and facilitate their colonization within the host to evade in-vivo immune responses and antibiotic attack. In addition, intracellular bacteria, such as *Listeria and Mycobacteria*, which evade other immune mechanisms by replicating within phagocytes, are able to successfully manipulate host cell pathways and use them as a basis for reproduction, dissemination, and persistence during infection.

T-cell-based cellular immunity plays a key role in the immune mechanism of intracellular bacterial clearance (Lewinsohn et al., 2011). CD4/CD8 cytotoxic T lymphocytes (CTLs) have been shown to contribute to host defense against intracellular pathogens (Canaday et al., 2001; Doedens et al., 2013; Murray and Mckay, 2021). TCR activates CTLs after recognizing the antigen peptide-major histocompatibility complex (MHC) complex and then proliferates and differentiates. These cells migrate to all corners of the body to clear the infection and generate a specific immune response. This review focuses on the mechanisms of CTLs differentiation and pathways to kill intracellular bacteria. This review could provide new insights into the effector function of CTLs.

## 2 Biological characteristics of CTLs

### 2.1 Development of T cells

T cells are derived from pluripotent hematopoietic stem cells (HSCs) in the bone marrow (Flippe et al., 2020). They first differentiate into lymphoid progenitor cells in the bone marrow and then enter the thymus through the blood circulation. T cells undergo positive and negative selection in the thymic cortex and medulla, respectively (Starr et al., 2003). In positive selection, T cells which bind moderately to MHC complexes receive the survival signals which determines if the T cells will become CD8^+^ or CD4^+^ single positive (SP) T cells. MHC-I-specific TCR signals generate CD8 SP T cells while MHC-II-specific TCR signals generate CD4 SP T cells. T-cell tolerance is achieved through the mechanism of negative selection in the thymic medulla which deleted self-reactive T cells. T cells that interact to the autoantigenic peptide-MHC molecular complexes would be killed in the negative selection process. In addition, T cells undergo the V and DJ segments rearrangement of T cell receptors (TCRs) TCRα and TCRβ genomic loci in early T cell precursors (Rytkönen-Nissinen et al., 1999) to enable the acquisition of TCR diversity and the ability to recognize various antigens in nature. The CD8 SP and CD4 SP cells are positively selected to differentiate into functionally distinct subpopulations, i.e., cytotoxic and Th (helper T cells). Recent studies have shown that CD4^+^ T cells also have the ability to acquire cytotoxic activity (Suni et al., 2001; Appay et al., 2002; Brown, 2010; Patil et al., 2018).

### 2.2 Differentiation of T cell precursor to naive T cells

CD4/CD8 CTLs are differentiated from CD4^+^ CD8^+^ T cell precursors. These differentiations are regulated by key transcription factors. Expression of the Th-induced BTB/POZ domain-containing Kruppel zinc-finger transcription factor ThPOK (cKrox, encoded by Zbtb7b) and Runt-related transcription factor 3 (Runx3) is essential for differentiation into CD4 SP and CD8 SP cells, respectively. Then, CD4 SP and CD8 SP cells differentiate into helper and cytotoxic lineages, respectively. The antagonism between these two transcription factors is a key mechanism for differentiation into two cell lineages. ThPOK inhibits the cytolytic program of MHC class II-restricted CD4^+^ T cells and Runx3. Meanwhile, Runx3 inhibits the expression of ThPOK and promotes the acquisition of cytotoxicity (Taniuchi, 2018; Knudson et al., 2021). CD4^+^ T cells retain the potential to become CD4 CTLs after exposure to a specific environment, which inhibits ThPOK, and upregulate the expression of Runx3, leading to the acquisition of cytotoxic activity. Mature CD4^+^ Th cells could also terminate ThPOK transcription by a transcriptional switch in the ThPOK silencer, turning off ThPOK expression and converting themselves into cytotoxic MHC class II effectors, thereby acquiring cytolytic capacity. And the mechanism regulating ThPOK silencer activity is a key to determining lineage-specific THPOK expression and should be a nuclear target of TCR signaling (Taniuchi, 2018). Besides ThPOK and Runx3, the transcription factor GATA3 is important in early T cell lineage differentiation, acting upstream of ThPOK, and its downregulation promotes the acquisition of cytolytic capacity by CD4^+^/CD8^+^ T cells (Shan et al., 2017). POZ/BTB and AT-hook-containing zinc finger protein 1 (PATZ1/MAZR) negatively regulates ThPOK expression (Mucida et al., 2013).

**Figure 1:**
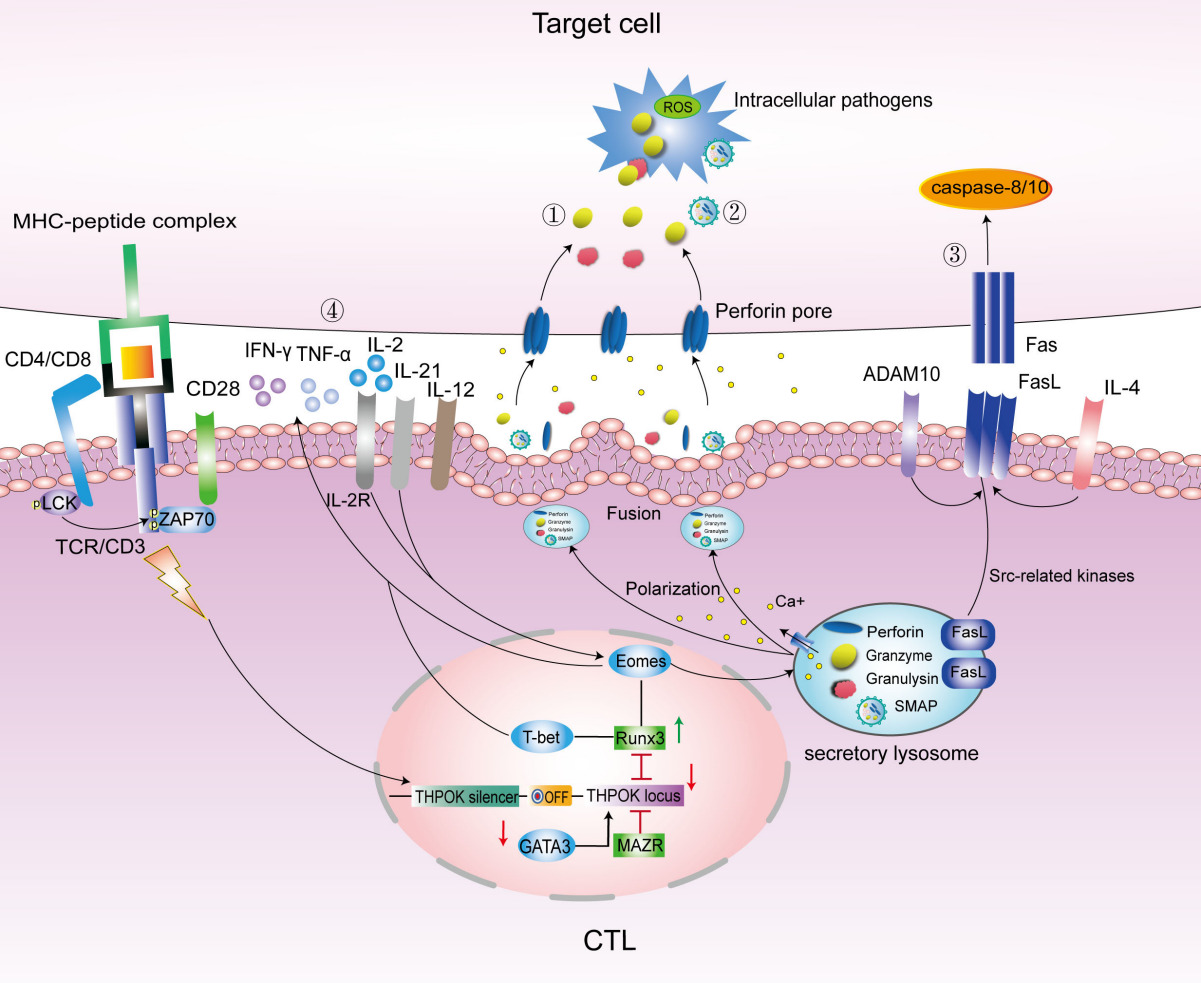
Main pathways to kill intracellular bacteria by CTLs. Under the regulation of transcription factors GATA3/THPOK/Runx3, CD4/CD8 T cells differentiate into CD4/CD8 CTLs. Eomes and T-bet promote the secretion of cytotoxic granules contents PFN, Gzm and GNLY, and IFN-γ to kill target cells. The main pathways to kill intracellular bacteria included the following: ①.PFN/GNLY/GzmB-mediated cytotoxic granules pathway; ②.SMAPs pathway; ③. Fas-FasL cell apoptosis pathway; and ④. Cytokine pathway mainly include IFN-γ, TNF-α, IL-12, IL-21and IL-4. LAMP, lysosome-associated membrane glycoprotein; ThPOK, T-helper inducing POZ-Kruppel-like factor; Runx3, Runt-related transcription factor 3; Eomes, Eomesodermin; MAZR, POZ/BTB and AT-hook-containing zinc finger protein 1; ADAM10, A Disintegrin And Metalloproteinase 10; PFN, perforin; Gzm, granzyme; FasL, Fas ligand; SMAPs, supramolecular attack particles.

IL-2 signaling is essential for the induction of CTLs. IL-2 induces the expression of the transcription factor Eomesodermin (Eomes), which promotes the expression of IFN-γ and cytotoxic granules. The transcription factor T-bet induces CTLs differentiation (Juno et al., 2017) and the expression of granzyme B (GzmB) and perforin (PFN). Thus, CTLs may be regulated by T-bet and Eomes. CRTAM^+^-activated CD4^+^ T cells cultured with IL-2 could efficiently express Eomes and produce CD4 CTLs (Juno et al., 2017), as shown in **Figure 1**.

### 2.3 Cytotoxic properties of CTLs

#### 2.3.1 Cytotoxic properties of CD8 CTLs

CD8^+^ T cells mainly recognize endogenous antigens on MHC class I molecules, which are expressed on almost all cells. Exogenous antigens are mainly presented on MHC class II molecules. However, some exogenous antigens, such as bacteria, fungi, and parasites, could also be cross-presented to CD8^+^ T cells by some professional antigen-presenting cells (APCs) *via* the MHC class I molecules, including dendritic cells (DCs) and some phagocytes. Classical type 1 DCs (cDC1s) are considered the major cross-presenting cells, which are dependent on the transcription factor Batf3 (Enders et al., 2020). APCs internalize and process antigens, producing immunogenic peptides that enable antigen presentation to T cells, which provide the signals that trigger T cell activation, proliferation, and differentiation into CTLs (Saiz et al., 2018).

CTLs express a large number of tissue-homing molecules and chemokine receptors, and such expression enables CTLs to migrate to nonlymphoid tissues or to sites of inflammation (Butcher and Picker, 1996). Then, CTLs make direct contact with target cells by formation of a lytic immunological synapse (IS) (Dustin and Long, 2010; Basu and Huse, 2017). CD8 CTLs release cytotoxic granules contents PFN, Gzm, and granulysin (GNLY) and supramolecular attack particles (SMAPs), a newly discovered cytotoxic multiprotein complex containing PFN, GzmB, and other substances. These granules enter the interior of target cells from the IS site to kill intracellular bacteria; induce apoptosis *via* Fas ligand (FasL); and produce some cytokines, such as IFN-γ and TNF-α, to attack target cells. After antigen clearance, a large number of effector CD8 T cells die, leaving a small number of “memory” CD8^+^ T cells for rapid immune response to the next invasion of the same antigen and timely clearance of the target cells.

#### 2.3.2 Cytotoxic properties of CD4 CTLs

CD4^+^ T cells recognize antigenic peptides linked to MHC class II molecules. MHC-II-restricted cytotoxic T cells were first discovered decades ago in allogeneic responses (Wagner et al., 1975; Billings et al., 1977). Since 2001, the ability of human CD4^+^ T cells to function as a CTLs *in vivo* has also been widely reported (Suni et al., 2001; Appay et al., 2002; Brown, 2010; Patil et al., 2018). A study showed that in *MTB*-infected subjects, heparin-binding hemagglutinin (HBHA) induced CD4^+^ T cells degranulation with concomitant production of IFN-γ, PFN, Gzm, and GNLY. This finding suggested that HBHA induced a subpopulation of CD4^+^ T cells with lytic functions that possessed Th1 and cytotoxic properties (Aerts et al., 2019). Hildemann et al. demonstrated that CD4 CTLs could be readily generated using in-vivo CTLs assays and have comparable CTLs activity to CD8 CTLs when adjusted for factors, such as effector–target ratio (Hildemann et al., 2013). CD4 CTLs surface markers could also be used to label CD8 CTLs with cytotoxic function-related markers, such as lysosome-associated membrane glycoproteins (LAMPs) [e.g., LAMP-1 (CD107a) and LAMP-2 (CD107b)]. (Takeuchi and Saito, 2017). Immune cells contain membrane-bound organelles called “secretory vesicles,” “secretory lysosomes (SLs)” or “granules” (Basu et al., 2016). Upon contact with an invading pathogen or receiving signals from the pathogen or surrounding cells, the organelles move to the cell surface where they fuse with the plasma membrane and release their contents during degranulation through complex signal transduction. Such molecules like LAMP are markers of degranulation and abundantly expressed on the cell surface. In addition, they could also be marked with class I restricted T cell-associated molecule (CRTAM), which is also an early activation marker of CD8 CTLs. These shared markers suggest that CD4 CTLs have similar characteristics and functions to CD8 CTLs, are able to secrete PFN and GzmB, and could kill target cells.

However, CTLs are heterogeneous in the expression of these proteins. A study found three major subpopulations of CTLs: monocytotoxic T lymphocytes (M-CTLs) expressing only GzmB; dicytotoxic T lymphocytes (D-CTLs) expressing PFN and GzmB; and T-CTLs expressing all three proteins GzmB, PFN, and GNLY. This study also found that the frequency of T-CTLs in leprosy was the greatest in a group of patients able to restrict the infection. This result suggested that T-CTL is a major effector T cell in the control of leprosy infection (Balin et al., 2018). T-CTLs express the NK receptor KLRC2 (NKG2C) on its surface. Triggering NKG2C could activate CD8 CTLs in the absence of anti-CD3 stimulation to release cytotoxic granule proteins in a TCR- and antigen-independent manner, whereas CD4 CTLs execute their cytotoxic function in an antigenic peptide-dependent and MHC class II-restricted manner. Antigenic peptide-loaded MHC class II molecules are only expressed on the surface of professional APCs. The intracellular bacterial killing activity of CD4 CTLs is thus targeted.

## 3 Mechanisms of killing intracellular bacteria by CTLs

Mature T lymphocytes specifically recognize antigens presented by APCs through TCR, and transduce the activation signal generated by TCR-recognized antigens through the TCR-CD3 complex and CD28-mediated co-stimulation (signal 2) and cytokine IL-2 to induce T cell activation and proliferation. After an antigen is recognized, T lymphocytes proliferate and differentiate into effector T cells, including CD4 CTLs and CD8 CTLs. As mentioned earlier, CTLs could exert their intracellular killing effect through the following four pathways (**Figure 1**).

### 3.1 PFN/GNLY/GzmB-mediated cytotoxic granules pathway

CTLs release soluble cytotoxic proteins PFN, GZM, and GNLY into the lytic IS and initiate multiple pathways, leading to target cells death (Froelich et al., 1996; Keefe et al., 2005). IS a structural domain of an adhesion protein ring surrounding an internal signaling molecule. The lytic IS is formed when CTLs recognize target cells through the TCR and establish direct contact with target cells (Soares et al., 2013). When cytotoxic granules contents enter the lytic IS, PFN perforates the plasma membranes of their target cells and Gzm and GNLY enter the target cells. The cytosolic exocytosis of granules is regulated by intracellular free Ca^2+^ (Reddy et al., 2001). CTLs recognize the target and generate a signaling event that leads to a pronounced increase in Cytosolic Ca^2+^ concentration. Lysosomal Ca^2+^ release regulates lysosomal trafficking and exocytosis and promotes PFN degranulation and efflux (Li et al., 2019).

**Figure 2:**
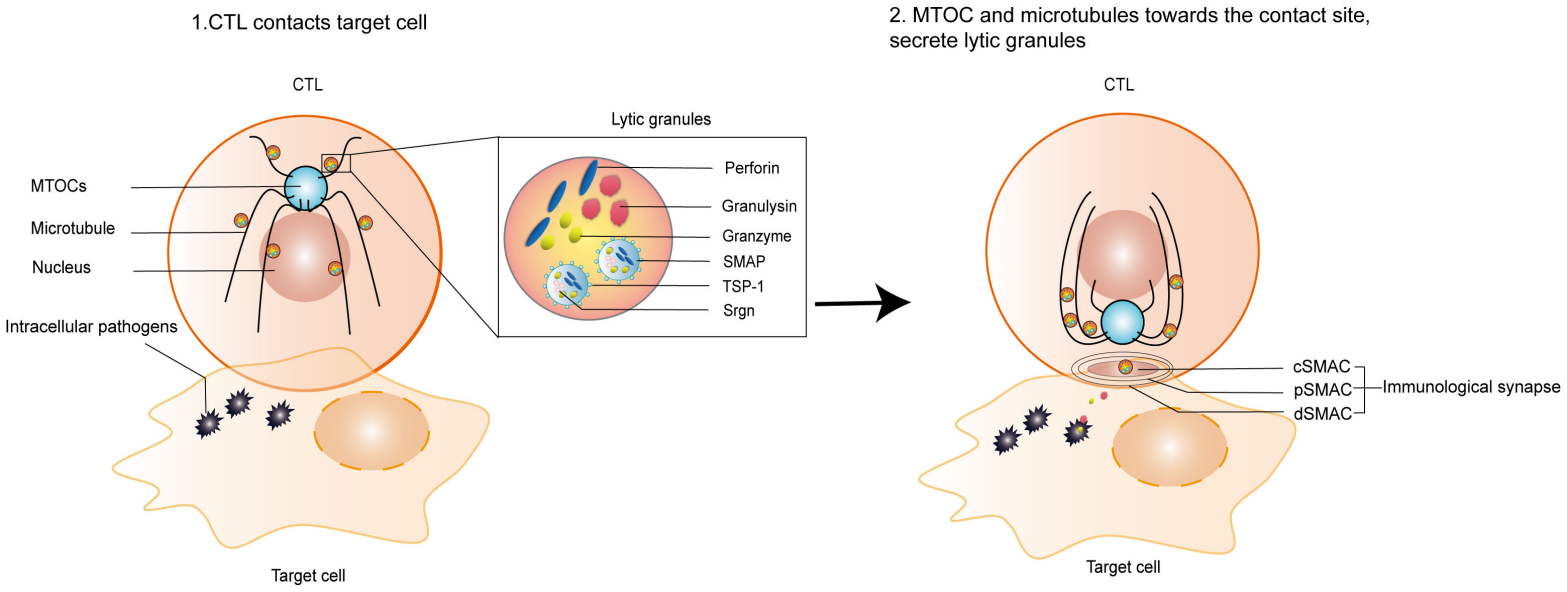
Formation of lytic IS and release of lytic granules. 1. CTLs contact infected target cells; 2. Formation of a lytic IS between CTLs and infected target cells, orienting the MTOC and the entire MTs network towards the contact sites where the lytic IS is being organized. Lytic granules, such as PFN, Gzm, GNLY, and SMAP, are released onto the surface of target cells or into target cells to kill intracellular bacteria. IS is composed of central supramolecular activation clusters (cSMACs), peripheral supramolecular activation clusters (pSMACs), and distal supramolecular activation clusters (dSMACs). SMAP includes a glycoprotein shell named TSP-1 and a core containing PFN, GzmB and Srgn cytotoxic proteins. IS, immunological synapse; MTOCs, microtubule organizing centers; MTs, Microtubules.

IS has a central TCR-MHC interaction cluster surrounded by an LFA-1-ICAM-1 adhesion loop and a distal LFA-1-ICAM-1 adhesion loop, including the transmembrane tyrosine phosphatase CD45. Kupfer referred to these structures as supramolecular activation clusters (SMACs) and divided them into central, peripheral, and distal regions or central supramolecular activation clusters (cSMACs), peripheral supramolecular activation clusters (pSMACs), and distal supramolecular activation clusters (dSMACs) (Dustin, 2014). cSMAC containing ligand-bound antigen receptors and associated signaling proteins serves as a hub for signal transduction and active secretion. pSMAC is rich in adhesion molecules, and it serves to promote adhesion between CTLs and target cells. dSMAC is enclosed by a dense ring of filamentous actin (F-actin). The actin cytoskeleton of dSMAC exerts mechanical forces on synapses to enhance and direct cytotoxic killing (Basu and Huse, 2017). Microtubules (MTs) and microtubule organizing centers (MTOCs) coordinate the structural and transport functions in cells (Wu and Akhmanova, 2017; Sallee and Feldman, 2021). CTLs orient their MTOCs and the entire MT network, reorienting them towards the contact sites where IS is being organized (**Figure 2**). SLs are organelles in CTLs that store a wide range of proteases and membrane proteins (Peters et al., 1991; Voskoboinik et al., 2010). SLs contain lysosomal hydrolases that act at acidic pH and unique proteases that act at neutral pH, including cytotoxic granules contents, such as PFN, Gzm, and GNLY (Burkhardt et al., 1990). The mediators of CTLs-mediated killing have been identified as specialized SLs known as lytic granules (LGs). LGs are hybrid organelles that specialize in the secretion of cytotoxic effector molecules. In addition to storing cytotoxic proteins, LGs function as conventional lysosomes. These granules are generally harmless to CTLs, but they have a lethal killing effect on target cells. CTLs degranulate and secrete PFN, Gzm, and GNLY. Cytotoxic granules move along MTs towards the target cells (Stinchcombe and Griffiths, 2007) and then transport from the MTOC to the plasma membrane at the synapse (de la Roche et al., 2016). Finally, they enter the target cells cytoplasm through the small secretory cleft formed between the CTLs and the target cells inside.

PFN is a granule protein that forms transmembrane pores in target cell membrane under neutral pH and Ca^2+^ conditions. LGs with acidic pH are a safe storage chamber for PFN. GNLY is a protein present in the cytotoxic granules of CTLs. GNLY could induce discrete lesions and distortions in the bacterial surface (Stenger et al., 1998). So, GNLY may play an important role in antibacterial activity by damaging or penetrating the bacterial cell wall to allow GzmB to enter and cleave the bacterial substrate (Walch et al., 2015). PFN and GNLY were co-localized in cytotoxic granules. CD4^+^T-cell lines expressed GNLY more frequently than PFN, whereas CD8^+^T-cell lines expressed PFN more frequently than GNLY (Ochoa et al., 2001). GzmB is a serine protease that could degrade intracellular microbial defense against oxygen radicals. And GzmB was released in its soluble form or as SMAP (Liu et al., 2020). Studies have shown that PFN formed pores of 16–22 nm in inner diameter (Baran et al., 2009) on the surface of target cells. Then, GzmB (approximately 4 nm in diameter) and GNLY diffused across the pores formed by PFN into the cytoplasm of target cells. Finally, Gzms enter the bacteria by the action of GNLY. A study labeled Gzms and GNLY with fluorescent AlexaFluor (AF)-488 and AF-647, respectively. Then, they treated *E. coli* with AF-488 Gzms in the presence or absence of AF-647 GNLY. Confocal microscopy showed that Gzms were internalized into bacteria in a GNLY-dependent manner; GzmB entered the bacteria, whereas GNLY stayed on the surface (Walch et al., 2015). The study further showed that bacteria were less viable and abundant in the presence of all three proteins (PFN, GzmB, and GNLY) than those in which two of them were used. Overall, PFN, Gzms, and GNLY work together to enter target cells to kill intracellular bacteria.

After GzmB enters intracellular microorganisms, the death of these microorganisms is mediated by superoxide. GzmB generates ROS through proteolysis (Jacquemin et al., 2015). Scavenging of superoxide anion or overexpression of superoxide dismutase or peroxidase *in vitro* and *in vivo* inhibited the death of intracellular microorganisms, indicating the importance of oxidative damage in intracellular microbial killing. Gzms also break down bacteria-produced superoxide dismutase and catalase that inactivate superoxide anions and peroxides, thereby enhancing bacterial vulnerability to oxidative damage and promoting bacterial death. The mechanism of cytolytic granule secretion in CTLs works very rapidly. TCRs aggregate centrally to form cSMAC within 1 minute, around mobile centrosomes within 2.5 minutes, and reach the synaptic plasma membrane after 6 minutes (Ritter et al., 2015). *In vitro*, lysis of target cells takes only a few minutes (Wiedemann et al., 2006).

PFN and Gzms secreted by CTLs play an important part in controlling *MTB* and *Listeria* infection. Studies showed that the number of CD8 CTLs (Serbina et al., 2000) and the expression of Gzm, GNLY, and PFN (Canaday et al., 2001) increased in the lungs and lung draining lymph nodes of mice infected with *MTB*. Among them, PFN is required for *MTB*-specific CD8 CTLs cytotoxic activity (Woodworth et al., 2008). PFN-deficient CD8 CTLs have a reduced cytolytic capacity *in vivo*. The lytic activity of CTLs progressively decreases with disease progression (De La Barrera et al., 2003).The adaptive immune response induced by *Listeria* is mainly mediated by CTLs (Zenewicz and Shen, 2007). When PFN and GNLY were present together, the induced intracellular *Listeria* lysis remarkable increased (Walch et al., 2007). When the CTLs cytolysis rate of infected cells exceeded the bacterial dissemination rate, PFN showed a substantial effect on bacterial clearance and slowed direct cellular spread of intracellular *Listeria* (San Mateo et al., 2002). Clearance of *Listeria* was also delayed in PFN-deficient mice. GNLY did not provide a significant survival benefit in either CD4^+^ T cell or CD8^+^ T cell-depleted mice of parasite infection (Dotiwala et al., 2016).

### 3.2 SMAPs pathway

SMAPs are autonomously cytotoxic multiprotein complexes and approximately 120 nm in diameter. Their structure contain a glycoprotein shell of thrombospondin-1 (TSP-1), an extracellular matrix Ca^2+^-binding glycoprotein involved in cell–cell and cell–matrix interactions (Adams and Lawler, 2011), and core substances PFN, GzmB, and Serglycin (Srgn) (Bálint et al., 2020), as shown in **Figure 2**. Srgn is expressed by CTLs and other innate immune cells, such as macrophages and neutrophils. It contributes to proper particle storage and promotes granule integrity (Pejler et al., 2009). The c-terminal 60-kDa structural domain of TSP-1 containing a Ca^2+^-binding repeat is essential for SMAPs assembly and their cytotoxic function. Srgn, GzmB, and PFN coexist as multimeric complexes within SLs (Metkar et al., 2002). These particles are stored in the SLs of CD8 CTLs and released to the target cells membrane *via* IS upon CD8 CTLs contact with target cells. SMAPs remained attached to the supported lipid bilayers after CD8 CTLs removal, released cytotoxicity, and caused target cells death independently (Rettig and Baldari, 2020). In another study, synaptobrevin2-mRFP knocked-in mice were used to isolate fusogenic cytotoxic granules, and two classes of fusion-competent granules, with different diameters, morphologies, and protein compositions were identified, referred to as single core granules (SCG) and multi core granules (MCG). SCG releases soluble GzmB. Meanwhile, MCG could be labelled with TSP-1 in SMAPs. Fusion of SCG and MCG releases intact SMAP into the target cells to kill them (Chang et al., 2022). SMAPs are a new class of mixed particles that share their cytotoxic components with LGs, thus having similar soluble capacity. However, SMAPs have a unique glycoprotein shell of TSP-1, whereas other cytotoxic particles have phospholipid membranes on their surface. The mechanism of SMAPs entry into the target cells has not been clarified.

### 3.3 Fas-FasL cell apoptosis pathway

Binding of Fas to FasL (CD95-CD95L) leading to apoptosis of target cells is another pathway of CTLs-mediated cytotoxicity (Ivanova et al., 2017; Sharapova et al., 2017). The CTLs-induced Fas-FasL apoptosis pathway is a Ca^2+^-independent/PI3K-dependent pathway (Genolet et al., 2014). The Fas molecule is a cell surface protein, weighing 45 kda, and belonging to the tumor necrosis factor receptor (TNFR) type I. Fas binds to FasL *via* cysteine-rich extracellular domain. FasL is a 40-kda type II transmembrane protein of the TNF family, mainly expressed in activated T cells. It is stored in the outer membrane of the cytoplasmic granules of CTLs, whereas PFN is localized to the inner vesicles of the cytoplasmic granules (Kojima et al., 2002). Fas is expressed only when myeloid cells, such as macrophages or lymphocytes, are activated. During cytoplasmic granule degranulation, these molecules are transferred to the cell surface when the outer membrane of the cytoplasmic granule fuses with the plasma membrane (He et al., 2010). During this process, the release of FasL is regulated by A Disintegrin And Metalloproteinase 10 (ADAM10) (Lettau et al., 2008). ADAM10 sheds the FasL and thereby regulates T cell activation and effector function. During TCR/CD3/CD28 stimulation, kinases associated with Src are the basis for initiating the signaling cascade leading to FasL shedding, and Src-related kinases were identified as putative interactors and regulators of ADAM proteases. ADAM10 and FAS are in the same location in SL. Knockdown of ADAM10 reduces CD3/CD28-induced FasL shedding (Ebsen et al., 2015).

FasL is induced on the cell surface of CTLs-stimulated activation through TCR, co-stimulatory molecules, and cytokine receptors. Purified FasL could lyse Fas-expressing cells. Upon recognition of target cells, the FasL on the surface of CTLs could be released extracellularly to bind with Fas on the surface of target cells; leading to crosslinking of the Fas cell membrane “death zone”; conducting death signals into the cell; inducing the replenishment and activation of apoptosis-initiating proteases, such as caspase-8 and caspase-10 (Yamada et al., 2017); and causing the apoptosis of target cells with the intracellular bacteria through various pathways. Fas-FasL-mediated cytotoxicity is highly specific and directional, targeting only antigen-containing target cells and not killing bystander cells (Kojima et al., 1994). The target cells membrane of Fas-induced apoptosis remains intact for a considerable period of time, and its target cells lysis is slightly slower than PFN-mediated target cells lysis (Hahn et al., 1995).

Fas-FasL cell apoptosis mediated by CTLs plays an important role in controlling *Chlamydia* and *Listeria* infection. The obligate intracellular parasite *Chlamydia* infection induces a CTLs response at the site of infection (Kim et al., 2000), and when Fas-associated proteins with a death domain directly activate caspase-8, *Chlamydia trachomatis* fails to inhibit apoptosis (Waguia Kontchou et al., 2016).The expression of Fas on the surface of CTLs increases with time after *Listeria* infection. The Fas^+^ population accounted for 53% and 64% of CD4 and CD8 T cells in infected mice, respectively, compared with 33% and 36% in normal mice, respectively (Yang et al., 2014). Bacterial CFU counts were found to be increased in the liver and spleen of Fas-deficient mice, and Fas deficiency severely affected CD8 CTLs lysis of *Listeria*-infected hepatocytes. The study suggested that Fas and PFN have complementary roles in host defense against intracellular bacterial pathogens (Jensen et al., 1998).

### 3.4 Cytokine pathway

IFN-γ, TNF-α, IL-12, IL-21, IL-4, and other cytokines play an instrumental role in the protective immune response of CTLs to clear intracellular bacteria. CTLs secrete cytokines, such as IFN-γ, TNF-α, and IL-2, after infection. IFN-γ could induce nitric oxide synthase, which causes macrophages to produce nitric oxide, a powerful microbicidal molecule. These cytokines enhance the killing effect against intracellular bacterial infection by activating macrophages or enhancing the immune effect of CTLs. IL-12 is an important Th1 cytokine which enhances CTLs effector function. It is also an important third signal cytokine for full activation of CTLs in murine models (Schurich et al., 2013). The IL-12-stimulated exosomes of CTLs directly activates bystander naive CD8 T cells to produce IFN-γ and GzmB (Li et al., 2017). IL-21 is produced by CD4^+^ T cell subsets, and it regulates the expression of transcription factor Eomes enhances CD8 CTLs expansion and effector functions (Cheekatla et al., 2017). It also plays a part in the control of bacterial infection (Booty et al., 2016). IL-4 is secreted by Th2 cells. IL-4 increases FasL expression on T cells, leading to a shift in the CTLs killing mechanism from a PFN-mediated cytolytic pathway to a FasL-mediated cytolytic pathway (Aung and Graham, 2000).

In *Chlamydia trachomatis* infection, CTLs produce protection through IFN-γ to control *C. trachomatis* infection (Lampe et al., 1998; Gondek et al., 2012). *Mycobacterium avium* intranasal infection in mice induces the proliferation of CD4 and CD8 CTLs and the production of protective IFN-γ. CTLs also suppress infection by producing IFN-γ and activating macrophages. The *Salmonella* genus is the most common foodborne pathogen. *Salmonella* can evade various extracellular immune responses by triggering its own phagocytosis through macrophages (Thompson et al., 2011) and being encapsulated in *Salmonella*-containing vesicles SCV (Wang et al., 2020). *Salmonella enterica* causes persistent intracellular infection while stimulating IFN-γ-producing CD4^+^ T cell responses (Goldberg et al., 2018), which, in turn, could produce IFN-γ to control phagosome infection and then bind to IFN-γ receptors on nearby infected phagocytes (Müller et al., 2012). After infection of germ-line NR2F6-deficient mice with *Listeria*, CD8 CTLs enhanced antigen-specific immune memory cell formation and inflammatory cytokine secretion, such as IFN-γ, TNF-α, and IL-2. After re-infection, the IFN-γ response was remarkable enhanced, the clonal expansion was increased, the host antigen-specific CD8 CTLs response was enhanced, and the memory effect formation was established in the early stage of the antibacterial immune response and mediated by IFN-γ (Jakic et al., 2021). IL-12 induces the expression of IFN-γ and activates antigen-specific lymphocytes capable of producing protective granulomas against *MTB* (Cooper et al., 1997).

## 4 Research progress of CTLs in disease treatment

Drug resistance induced by intracellular bacteria becomes a problem in the treatment of their infections. Developing safe and effective vaccines to address the increasing challenges of these drug-resistant strains is an urgent issue (Lin et al., 2021). CTLs play an important role in the clearance of intracellular bacteria; thus, the development of CTLs-oriented vaccines may be a feasible and promising therapeutic approach to control intracellular bacterial infections (Reinherz et al., 2014).

A study developed three tuberculosis (TB) therapeutic vaccines, HVJ-Envelope/HSP65+IL-12 DNA vaccine (HSP65-vaccine), granulysin vaccine and killed specific secretory protein at 37 kDa (Ksp37) vaccine. These vaccines showed therapeutic effect against TB infection in mice. The combination of HSP65-granulysin vaccine had a synergistic therapeutic effect. Granulysin and Ksp37 vaccine both augmented differentiation of CTLs against TB (Kita et al., 2013). DNA vaccines could induce various humoral and cellular immune responses. Bacille Calmette-Guérin (BCG)-prime DNA-booster vaccination strategy has been shown to induce greater protection against TB than BCG alone. DNA vaccines have been observed to cause strong CD8 CTLs-mediated immune responses (Bruffaerts et al., 2014). BALB/c mice immunized with a ubiquitin-fused Mycobacterium antigen-encoded DNA vaccine showed potent enhanced specific cytotoxicity and increased specific IFN-g-producing CD8 CTLs (Delogu et al., 2002). A randomized, controlled, double-blind crossover clinical study from the United States demonstrated that the oral live *typhoid* vaccine CVD908-htrA could induce strong antibody responses against soluble *typhoid Bacillus* Ags and the production of IFN-γ. The effect of elicited specific CD8 CTLs *typhoid* immune responses was also further assessed. CTLs were detectable within 14 days after immunization, this immune response persisted up to day 56, and a significant correlation was found between CTLs and IFN-γ release (Salerno-Gonçalves et al., 2003). A developed novel T-cell oriented vaccine is composed of long peptides and poly lactic-co-glycolic acid (PLGA) microparticles, against intracellular bacteria. The vaccine could promote antigen cross-presentation and induce strong CTLs response, and it has an effective protective effect on intracellular bacteria (Tanaka et al., 2020). A study identified novel CTLs epitopes from *MTB* efflux pumps Rv1258c and Rv1410c. The natural peptide Rv1410c-p510 and its analogs could induce potent T cell responses and efficiently lyse peptide target cells. The CTLs epitopes discovered in this study may serve as candidate genes for future multivalent peptide vaccines against drug-resistant *MTB* (Zhu et al., 2011). Another study constructed a novel recombinant *Mycobacterium smegmatis* (rMS) strain that expresses Ag85B and ESAT6 fusion protein (AE–rMS), which could produce IFN-γ and IL-2 on mice, thus increasing antigen-specific CTLs activity. The treatment of AE-rMS combined with anti-TB drugs isoniazid and rifampicin could further reduce bacterial load and improve lung pathological damage, thereby providing an effective therapeutic strategy for the treatment of TB (Wang et al., 2014).

## 5 Conclusion

PFN delivers cytotoxic proteases Gzm and GNLY to target cells through IS when killer lymphocytes recognize the infected cells. During this process, PFN breaks the membrane, and GNLY stays in the target cells membrane to help Gzm enter into target cells and intracellular pathogenic microorganisms. Only when PFN and GNLY coexist, Gzm could enter into intracellular pathogenic microorganisms and host target cells. Then, it produces superoxide anions and ROS, which decompose superoxide dismutase and catalase produced by bacteria that could inactivate superoxide anions and peroxides, thereby enhancing the bacterial response to oxidative damage and promoting bacterial death. In addition to releasing soluble cytotoxic granules to kill target cells and intracellular pathogenic microorganisms, CTLs could secrete cytotoxic multiprotein complexes with TSP-1 glycoprotein shells into target cells *via* IS. They could also promote apoptosis of target cells in a Ca^2+^-independent/PI3K-dependent manner through the binding of FasL on the surface of their own cells to Fas on the surface of target cells, rendering the death of intracellular pathogenic bacteria. They exert killing effect by secreting cytokines, such as IFN-γ and TNF-α. In the treatment of intracellular bacteria, the development of CTLs-oriented drugs or vaccines is an effective treatment method and idea. Researching the mechanisms of CD4/CD8 CTLs differentiation and effector functions is of great importance, especially for promoting the host immune response to clear intracellular colonizing bacteria and for antitumor and antiviral interventions and treatments.

## Author contributions

LT wrote and revised the manuscript. WZ, XW, ZH and LQ consulted the literatures and contributed with writing. HZ, XC, SZ and ZL revised the manuscript. All authors contributed to the article and approved the submitted version.

## Funding

This work was supported by National Natural Science Foundation of China (81873255, 82104834, 81403353), and Shanghai Hospital Development Center (SHDC2020CR2006A).

## Conflict of interest

The authors declare that the research was conducted in the absence of any commercial or financial relationships that could be construed as a potential conflict of interest.

## Publisher’s note

All claims expressed in this article are solely those of the authors and do not necessarily represent those of their affiliated organizations, or those of the publisher, the editors and the reviewers. Any product that may be evaluated in this article, or claim that may be made by its manufacturer, is not guaranteed or endorsed by the publisher.

## Abbreviations

CTLs, cytotoxic T lymphocytes; PFN, perforin; Gzm, granzyme; SMAPs, supramolecular attack particles; FasL, Fas ligand; HSC, hematopoietic stem cells; SP, single positive; TCR, T cell receptor; Th, helper T cells; ThPOK, Th-induced BTB/POZ domain-containing Kruppel zinc-finger transcription factor; Runx3, Runt-related transcription factor 3; Eomes, Eomesodermin; PATZ1/MAZR: POZ/BTB and AT-hook-containing zinc finger protein 1; DCs, dendritic cells; cDC1, class I classical DCs; IS, immunological synapse; GNLY, granulysin; HBHA, heparin-binding hemagglutinin; LAMP, lysosome-associated membrane glycoprotein; CRTAM, class I restricted T cell-associated molecule; M-CTLs, monocytotoxic T lymphocytes; D-CTLs, dicytotoxic T lymphocytes; APCs, antigen-presenting cells; PAMPs, pathogen-associated molecular patterns; PRRs, patternrecognition receptors; SMAC, Supramolecular activation clusters; cSMACs, central supramolecular activation clusters; pSMACs, peripheral supramolecular activation clusters; dSMACs, distal supramolecular activation clusters; MTs, Microtubules; MTOCs, microtubule organizing centers; MTB, Mycobacterium tuberculosis; TSP-1, thrombospondin-1; Srgn, Serglycin; SLs, secretory lysosomes; LGs, lytic granules; TNFR, tumor necrosis factor receptor; ADAM10, A Disintegrin And Metalloproteinase 10; TB, tuberculosis; HSP65-vaccine, HVJ-Envelope/HSP65+IL-12 DNA vaccine; Ksp37, killed specific secretory protein at 37 kDa; BCG, Bacille Calmette-Guérin; rMS, recombinant Mycobacterium smegmatis.
